# Impact of Individual-Level Social Capital on Quality of Life among AIDS Patients in China

**DOI:** 10.1371/journal.pone.0048888

**Published:** 2012-11-06

**Authors:** Ying Ma, Xia Qin, Ruoling Chen, Niannian Li, Ren Chen, Zhi Hu

**Affiliations:** 1 School of Health Service Management, Anhui Medical University, Hefei, China; 2 School of Public Health, Anhui Medical University, Hefei, China; 3 The First Affiliated Hospital of Anhui Medical University, Hefei, China; University of Ottawa, Canada

## Abstract

**Background:**

With growing recognition of the social determinants of health, social capital is an increasingly important construct in international health. However, the application of social capital discourse in response to HIV infection remains preliminary. The aim of this study was to assess the impact of social capital on quality of life (QoL) among adult patients with acquired immune deficiency syndrome (AIDS).

**Methods:**

A convenient sample of 283 patients receiving antiretroviral treatment (ART) was investigated in Anhui province, China. QoL data were collected using the Medical Outcomes Study HIV Survey (MOS-HIV) questionnaire. Social capital was measured using a self-developed questionnaire. Logistic regression models were used to explore associations between social capital and QoL.

**Results:**

The study sample had a mean physical health summary (PHS) score of 50.13±9.90 and a mean mental health summary (MHS) score of 41.64±11.68. Cronbach's α coefficients of the five multi-item scales of social capital ranged from 0.44 to 0.79. When other variables were controlled for, lower individual levels of reciprocity and trust were associated with a greater likelihood of having a poor PHS score (odds ratio [OR] = 2.02) or PHS score (OR = 6.90). Additionally, the factors of social support and social networks and ties were associated positively with MHS score (OR = 2.30, OR = 4.17, respectively).

**Conclusions:**

This is the first report to explore the effects of social capital on QoL of AIDS patients in China. The results indicate that social capital is a promising avenue for developing strategies to improve the QoL of AIDS patients in China, suggesting that the contribution of social capital should be fully exploited, especially with enhancement of QoL through social participation. Social capital development policy may be worthy of consideration.

## Introduction

Social capital has become a popular topic in public health research in recent years, though there has been a lack of consensus concerning its definition [1]–[4]. As reviewed recently by Murayama et al. [5], there are two distinct concepts of social capital in the literature. According to one conception, social capital represents the resources available to members of tightly knit communities. This interpretation could be described as the “social cohesion” definition which tends to emphasizes social capital as a group attribute. For example, Putnam considered social capital as “features of organization, such as trust, norms, and networks hat can improve the efficacy of society by facilitating coordinated actions” [6]. On the other hand, in the “networks” theory, social capital is defined in terms of resources that are embedded within an individual's social networks. For instance, Bourdieu regarded social capital as “the aggregate of the actual or potential resources which are linked to possession of durable networks” [6]. Within the health field, social capital has commonly been thought of from the “social cohesion” perspective, and this predilection persists to this day [5].

Social capital can also be defined at different levels, specifically at individual and collective levels [7].However, the individual-level social capital indexes are components of aggregated-level social capital [8]. Individual-level social capital offers the most simple and clearly defined units of measurement. Focusing on individuals avoids the common interpretation problems of analyses that stem from the use of aggregated data, in which the problem of the “modifiable area unit” may be encountered [9]. Moreover, decisions to invest in social capital are generally made by individuals, not communities [10]. For these reasons, we focused on individual-level social capital in the current study.

The construct of social capital used within health fields in China may differ from that in the West owing to cultural differences [7]. Famously, the Chinese people use “guanxi” (关系) or instrumental personal ties to acquire the resources they need [11]. Investments in social capital by way of developing social networks may provide individuals with access to resources and support [6]. Our systematic review of the relevant literature revealed that social capital is defined ubiquitously in accordance with the networks conception of social capital in the context of health care related studies. Thus, for this study, we have adopted the networks conception of social capital, as described by Meng and colleagues at Peking University, as our operational definition [6]. That is, social capital, in the current context, refers to networks of social relationships that may provide individuals and groups with access to resources and support. Within the context of this definition, we recognize that personal resources also include features of social structures (interpersonal trust and norms of reciprocity).

Although people generally have an intuitive understanding of quality of life (QoL) as a concept, it is still difficult to define it. The World Health Organization (WHO) has defined QoL as individuals' perceptions of their positions in life in the context of the culture and value systems in which they live and in relation to their goals, expectations, standards, and concerns. This definition focuses upon respondents' perceived QoL [12]. QoL is particularly relevant in research involving patients with acquired immune deficiency syndrome (AIDS). QoL measures have been used to evaluate the effectiveness of treatments and factors predicting the well-being of AIDS patients [13].

Despite the rapid accumulation of general population social capital studies, little attention has been paid to the utility of social capital by AIDS patients [14], [15]. Relative to the general population, AIDS patients are more likely to be socially isolated, more often diagnosed with chronic health conditions, and more likely to lack access to health care resources [14]. Furthermore, many AIDS patients experience chaotic personal environments that can lead to negative health outcomes [16]. Therefore, the association between social capital and health may be different among AIDS patients in comparison to the general population. Existing researches on social capital in relation to human immunodeficiency virus (HIV) have predominantly focused on the prevention of HIV transmission [17], namely, positive and negative effects of social capital on HIV prevention. For example, encouraging reductions in antenatal HIV prevalence from 30% to under 10% were observed between 1990 and 2005. Epstein noted that effective social mobilization, particularly through peer-to-peer networking, has been fundamental to this dramatic reduction in prevalence [18]. In the negative effect, Gregson et al. showed that young women participating in community groups had more risky lifestyles than those who were not participants [19]. To our knowledge, there have been no empirical studies on the impact of social capital on the QoL of AIDS patients in China or elsewhere.

In China, there were estimated 780,000 people living with HIV/AIDS and 133,524 AIDS patients, cumulatively, who received antiretroviral treatment (ART) by the end of 2011 [20]. Hence, the topic of QoL and social capital for AIDS patients is gaining importance. In this paper, we aimed to investigate the impact of social capital at the individual level on the QoL of AIDS patients.

## Methods

### Ethics statement

Ethical approval for the study was obtained from the Biomedical Ethics Committee, Anhui Medical University.

### Study setting

Anhui province, which is located in the southeast region of China, has a relatively low HIV/AIDS prevalence among Chinese provinces. In the 1990s, the primary cause of the AIDS epidemic in Anhui Province was illegal blood-collection, while in recent years sexual intercourse has become the main means of HIV transmission [21]. Based on the geographic distribution of AIDS patients in Anhui province, we selected one city in each of eight geographic areas: Maanshan city in eastern Anhui; Chuzhou city in northeastern Anhui, Benbu city in northern Anhui; Fuyang city in northwestern Anhui; Luan city in western Anhui; Anqing city in southwestern Anhui; Wuhu city in southern Anhui; and Langxi city in southeastern. From September 2010 to March 2011, cross-sectional surveys were conducted in these eight cities.

### Study population and data collection

This study examined a convenient selected sample, with the inclusion and exclusion criteria outlined below. Since 2003, the “Four free and One Care” policy has been enacted in response to the HIV epidemic in China [22]. All AIDS patients in China have been given the option to receive free ART. In fact, the percentage of patients who refuse to receive ART is very small; treatment coverage for AIDS patients in Anhui province was 93% in 2010 [21], namely, about one hundred AIDS patients refused to receive ART mainly because of identity exposure. Therefore, we excluded the AIDS patients who did not receive ART in respect of their confidentiality and because it was readily feasible to do so. To participate in our study, AIDS patients needed to meet the following criteria: (1) ≥18 years old; and (2) ART recipient for more than 1 month. The requirement of at least 1 month of ART was applied in order to minimize the influence of secondary drug effects on the patients' perceived QoL. According to the “National free AIDS antiviral medication manual” in China [23], in the first month of ART, patients are in an adjustment period in which they may experience new, unfamiliar drug side effects.

Trained investigators from the Anhui Medical University conducted face-to-face interviews with the patients with the support of staff at the local Center for Disease Control and Prevention (CDC). All of the eligible respondents were identified from the AIDS patient database of the local CDC. They were informed verbally via telephone of the purpose and procedure of the study, the confidentiality parameters, and the compensation for travelling expenses ahead of time. Study participants expressed a verbal understanding of these issues and signed consent forms. Most of the data collection was undertaken either in the local CDC or in the respondents' homes. Other information, such as CD4 count and the duration each individual has been living with HIV, was obtained from the patients' medical files in the local CDC. With an overall response rate of 90.52%, we conducted full interviews with a total of 283 participants: 23 in Maanshan city, 24 in Benbu city, 31 in Fuyang city, 32 in Langxi city, 47 in Luan city, 39 in Anqing city, 47 in Chuzhou city, and 40 in Wuhu city.

### Social capital measures

Social capital assessment was a small part of our survey and thus we did not administer an extensive social capital questionnaire. Based on our operational definition of social capital explained in the introductory text of this paper and in consideration of existing comprehensive instruments (e.g., the Word Bank′s Social Capital Assessment Tool) and the related literature [24], we selected some commonly used items and adapted them to the Chinese context.

Four dimensions of social capital were considered: social networks and ties; social support; social participation; and reciprocity and trust. Social networks and ties included the number of close relatives, the number of close friends, the relationship within one's neighborhood, and frequency of contact with the relatives, friends and neighbors. Social support mainly addressed moral and material support. Social participation involved the frequency of group and community participation. Reciprocity and trust was measured in terms of vertical trust (trust in hospitals, municipal authorities, etc.), horizontal trust (generalized trust in other people), and mutual support. This information is reported in Table S1.

Individual-level social capital was measured by producing a component score of each dimension using factor analysis which was grouped into a binary variable. The mean component score was used as the cutoff point: high individual-level social capital (component score ≥0) and low individual-level social capital (component score <0) [6]. Given that social capital is a multi-faceted concept, to prevent loss of important information, we performed analyses of each dimension separately.

### Socio-economic status (SES) and other risk factors

The general risk factors record contained: (1) socio-demographic information, including education level, main occupation, gender, family monthly income, current smoking and alcohol intake etc.; as well as (2) AIDS related information, including mode of transmission, duration of living with HIV, and CD4 cell count.

### Assessment of QoL

QoL was evaluated using the 35-item simplified Chinese simplified version of the Medical Outcomes Study HIV Health Survey (MOS-HIV) questionnaire [25]. The MOS-HIV, developed by Wu and colleagues, is one of the most widely used instruments for evaluating patients' clinical outcomes and their quality of life, which has been translated into various languages. Good psychometric properties of the questionnaire have been documented in different languages. The simplified Chinese version of the MOS-HIV questionnaire has previously been demonstrated to have good reliability and validity [22], [25].

The MOS-HIV measures 10 domains, including 8 multi-item domains (general health, physical function, role function, cognitive function, pain, mental health, energy/fatigue, and health distress) and 2 single-item domains (social function and QoL). We applied another single-item inquiry on health transition. Raw item scores were summed for each domain and transformed into a 0–100 scale, with higher scores indicating better functioning and well-being. Two summary scores, namely the physical health summary (PHS) score and mental health summary (MHS) score, were generated from the factor analysis of the 10 scales. We considered patients to have a poor quality of life if their PHS and/or MHS were at or below the 25th percentile of the distribution [26].

### Statistical analysis

A descriptive analysis was performed on the sample, and the results were expressed as means ± standard deviations (SDs), frequencies, and percentages. Using principal component analysis factoring for factor extraction, Cronbach's α values were calculated to evaluate the validity and reliability of social capital scale. Finally, a logistic regression was conducted to explore associations between social capital and QoL.

### Logistic regression

A logistic regression model was employed to calculate adjusted odds ratios (ORs) and 95% confidence intervals (CIs) and thereby reveal whether there was an association between each dimension of social capital and QoL, after controlling for demographic variables including gender, ethnicity, educational level, and marital status. All analyses were performed using the SPSS statistical package (Windows version 11.5, SPSS Inc., Chicago, Illinois), and p value <0.05 was taken as statistically significant.

## Results

### Descriptive statistics

Our study sample of 283 respondents had a mean age of 40.76±10.23 years (range, 18–70 years) and a mean CD4 count of 414.63±194.32 cells/mm^3^ (range, 189–763 cells/mm^3^). A full descriptive summary of the respondents is provided in Table 1. The respondents had mean PHS and MHS scores of 50.13±9.90 (range, 21.78–67.23) and 41.64±11.68 (range, 13.46–64.47), respectively. Nearly half, 44.2% had poor PHS scores and 32.4% had poor MHS scores. The detailed PHS and MHS data are reported in TableS2 as supplementary material.

**Table 1:** Characteristics of the study participants (N = 283).

Variables		No. persons	Percentage
Age group^a^	18–29 years	38	14.7
	30–39 years	74	28.6
	≥40 years	147	56.8
Gender	Male	161	56.9
	Female	122	43.1
Marital status	Unmarried	52	18.4
	Currently married	174	61.5
	Other (e.g. divorced, widowed)	57	20.1
Education level	Illiterate	53	18.7
	Primary	75	26.5
	Junior high	101	35.7
	Senior high+	54	19.1
Occupation	Farmer	65	23.0
	Laborer/merchant	37	13.1
	Carder/village/doctor/teacher	21	7.4
	Non-working	160	56.5
Ethnicity^a^	Han	253	97.7
	Other	6	2.3
Current smoker	No	186	65.7
	Yes	97	34.3
Current drinker	No	232	82.0
	Yes	51	18.0
Family monthly income (Yuan)	<1000	118	41.7
	≥1000	165	58.3
HIV transmission mode	Sharing needles	10	3.5
	Sexual relationship	178	62.9
	Blood	95	33.6
Duration living with HIV	<12 months	58	20.5
	≥12months	225	79.5

a missing = 24.

### Factor analysis and social capital characteristics

Four factors were extracted with eigenvalues above 1.0. After running a varimax orthogonal rotation, the four factors explained 64.5% of the total variance. Table 2 shows the factor loadings of all the social capital items. The results of the factor analysis were in good accordance with the original dimensions, with the exception that the item “Would you like to provide support for the residents in your community who need help?” was mainly explained by “social support” rather than by “reciprocity and trust”.

**Table 2:** Factor loading for each of 15 social capital items.

Items	Main Components			
	1	2	3	4
1. How many intimate relatives do you have?	**0.595**	0.211	−0.049	0.182
2. How many close friends do you have?	**0.760**	−0.025	0.087	0.072
3. How often do you visit your neighbors?	**−0.626**	−0.068	−0.137	−0.148
4. How often do you invite your neighbors to your home?	**0.756**	0.150	0.044	0.166
5. Can you get the care when you feel uncomfortable or are suffering from the disease flare-ups?	0.311	**0.797**	0.088	0.018
6. Can you get financial assistance when you experience family life difficulties?	0.518	**0.662**	0.130	0.034
7. Do you believe that if you have private problems, you can discuss them with residents in your community?	0.386	**0.585**	0.024	0.358
8. Who could you turn to for support when the above situation occurs?	0.017	**0.412**	0.167	0.083
9. How many groups or organizations have participated in?	0.220	0.156	0.089	**0.900**
10. How many times have you taken part in the activities held by organizations you have joined?	−0.218	0.133	−0.008	**0.717**
11. How many times have you participated in collective community activities?	0.054	0.08	0.035	**0.915**
12. Do you believe that the majority of residents in your community can be trusted?	−0.025	0.082	**0.932**	0.027
13. Do you believe that the majority of local hospital and CDC staff can be trusted?	0.079	−0.008	**0.346**	0.026
14. Do you believe that the majority of residents in your community participate in activities organized by the community for the benefit of only a few residents?	0.222	0.099	**0.910**	0.032
15. Would you like to provide support for residents in your community who need help?	0.443	**0.610**	0.221	0.076

The overall Cronbach's α coefficient for social capital was 0.75. The Cronbach's α coefficients of the four factors ranged from 0.44 to 0.79. The social networks and ties factor had the weakest internal consistency of the four factors (α = 0.44).

Individual respondent scores ranged from −2.00 to 2.46 for social networks and ties, from −3.38 to 1.92 for social participation, from −0.70 to 3.81 for reciprocity and trust, and from −3.22 to 2.44 for social support. The percentages of respondents with low individual-level social capital in the four dimensions were 49.5%, 78.4%, 40.6%, and 44.2%, respectively.

### Multivariate regression

#### PHS Score

Multivariate-adjusted ORs (Table 3) indicated that low individual-level reciprocity and trust was significantly associated with a higher likelihood of having a poor PHS score (OR = 2.02). With respect to SES, not drinking, having a low income (family monthly income <1000 Yuan), being 30–39 years old or ≥40 years old, and living with HIV for ≥12 months were significantly associated with increased risk of poor PHS.

**Table 3:** Social capital linked with poor PHS (N = 283).

Variables		OR (95%CI)	*p*
**Reciprocity and trust**	High individual level	1.00	
	Low individual level	**2.02 (1.06–3.82)**	**0.031**
Current drinker	Yes	1.00	
	No	2.90 (1.09**–**7.69)	0.032
Family monthly income (Yuan)	≥1000	1.00	
	<1000	4.02 (2.07**–**7.75)	<0.001
Age group (years)	18**–**29	1.00	
	30**–**39	3.68 (1.01**–**13.44)	0.049
	≥40	2.51 (1.17**–**5.41)	0.018
Duration living with HIV	<12 months	1.00	
	≥12months	2.51 (1.23**–**5.12)	0.012

ORs were adjusted for variables in the table, and further for gender, marital status, education level, occupation, ethnicity, current smoking status, CD4 count, and HIV transmission mode.

#### MHS Score

As shown in Table 4, low levels of social capital in the realms of reciprocity and trust, social networks and ties, and social support were significantly associated with a higher risk of a poor MHS score. With respect to SES, respondents who were illiterate and who had been living with HIV for at least 12 months had a higher probability of having a poor MHS score than those with more education or recent infection.

**Table 4:** Social capital linked with a poor MHS score (N = 283).

Variables		OR (95%CI)	*p*
**Reciprocity and trust**	High individual level	1.00	
	Low individual level	**6.90 (14.92–3.19)**	**<0.001**
**Social networks and ties**	High individual level	1.00	
	Low individual level	**4.17 (2.10–8.26)**	**<0.001**
**Social support**	High individual level	1.00	
	Low individual level	**2.30 (1.02–5.18)**	**0.046**
Education level	Illiterate	4.48 (1.50**–**13.37)	0.007
	Primary	0.87 (0.31**–**2.47)	0.797
	Junior high	0.83 (0.31**–**2.21)	0.715
	Senior high+	1.00	
Duration living with HIV	<12 months	1.00	
	≥12months	4.48 (1.55**–**12.90)	0.006

ORs adjusted for the same factors as those in Table 3.

## Discussion

Our study provides an initial exploration of correlations between the aspects of social capital and QoL among AIDS patients at the individual level in China. With further development, our findings can be used to develop evidence-based policy to improve the QoL of AIDS patients.

### Social capital and its measurement

The strengths of our analysis were that careful attention was given to the design and validation of the social capital questionnaire. We obtained better internal reliability values for the social capital questionnaire used in our survey (0.44–0.79) than the values obtained by previous studies conducted in mainland China [6], [27].

Somewhat surprisingly, for three of the four domains (networks and ties, reciprocity and trust, and social support, but not social participation), we found that participants had high individual-level social capital. Thus, our findings suggest that AIDS patients may not be as marginalized as previously thought [14]. However, more evidence on social capital among AIDS patients is needed before making strong conclusions in this regard. It should be noted that it is possible that since the participants were recruited with the help of CDC staff, they were already involved, at least to some degree, in their health care and through that involvement may have accessed supportive social services (e.g., Four Frees and One Care). Thus, involvement with the CDC and potentially referred social services may have led them to perceive government and community organizations as more trustworthy, and to feel that they were in contact with social resources. Thus the potential biasing influence of this factor is a limitation of this study.

### Social capital and QoL

Consistent with prior studies [28], [29], our analyses showed that high individual-level reciprocity and trust was associated with a lower probability of having poor PHS and MHS scores. Roberts et al. found that mutual trust between medical personnel and patients with HIV/AIDS is a key factor in the improvement of drug adherence, which enables ART to have optimal effectiveness [28]. More recently, Krause et al. reported that trust in one's providers for best possible care and trust in one's providers to protect privacy were significant predictors of functional QoL [29]. As noted above, the fact that the patients in our sample were receiving free ART provided by Chinese government may enhance their trust in social organizations and health service providers. This ART participation may also increase the patients exposure to health related information and allow them the opportunity to have any health concerns addressed as needed [30]. Thus, it will be interesting to tease apart the role that participation in these services may have on perceived social capital and QoL among patients living with AIDS.

We observed that one's level of social networks and ties was a significant predicator of one's mental health status, consistent with our expectations and previous research [31]–[33]. AIDS patients may gain emotional, material, and economic support from their social networks, which can increase hope, treatment adherence, and rapid diffusion of health information, which, in turn, would be expected to improve patients' QoL [34]. This finding suggests that interventions targeting improvement of QoL for AIDS patients may be enhanced by using or expanding existing social networks. Social capital has been used in other countries such as Rwanda, where those who want treatment must come to the clinic with a relative or member of their association [35].

Social support may provide a buffer against the adverse effects of stress caused by medical side effects, which may in turn increase individual well-being. Our finding that social support was associated with mental health but not physical health is in line with previous work by Bastardo et al. [36], but differs from recent findings by Yadav [35], who reported that social support associated significantly with both mental health and physical health. Further research is needed to probe the inconsistent association between social support and physical health.

The putative association between social participation and QoL is controversial [37]. In contrast to prior studies conducted in the West and in Africa [38], [39], we did not observe a significant association between social participation and QoL at the individual level. There are several possible reasons for the lack of such an association in our sample. Firstly, a relatively low percentage of our study participants reported group memberships. Group membership was originally developed in the Western literature as a factor intended to capture integration into civil society [40], which might affect health through such factors as dissemination of information. Formal organizations (e.g., neighborhood or parent-teacher associations, and community groups) are rare in China, though people may form informal groups that fulfill similar functions and lead to collective benefits. Our measures of membership may have under-estimated participation in these informal groups. Likewise, AIDS patients in China may have access to fewer non-government organizations than patients in western countries. Another possible explanation is that AIDS patients may worry that regular involvement in group activities may expose their personal lives. Thus, it may be prudent to modify the list of groups to more deeply examine whether there is an association between social participation and health in the future. Furthermore, our findings affirm that it is more appropriate to define social capital from the perspective of networks than from the social cohesion concepts.

### Limitations

Our study has some limitations. First, the analysis of the links between the different social capital variables was cross-sectional and hence cannot be used to conclude causal relationships. Second, the results may not be generalized to all Chinese AIDS patients. Our data were collected in Anhui province, which has a relatively low HIV/AIDS prevalence for China, and thus may not reflect the situation in other provinces due to regional differences in the epidemic characteristics of AIDS, prevention and control measures, funding, and policy environment. Finally, because we measured social capital at the individual level only, the impact of context-level social capital on QoL, and the interactive influence of individual-level and context-level social capital on QoL are not clear. These limitations notwithstanding, our study provides a base upon which future surveys examining the impact of social capital on the QoL of AIDS patients in the Chinese context can be built.

## Conclusions

As an exploratory study, it was not possible to obtain a truly representative sample of Chinese AIDS patients, but this limitation does not diminish the implications of our findings. Our study indicates that our self-developed social capital scale for Chinese AIDS patients has good reliability and validity, that a higher level of social capital is associated with a better QoL overall among AIDS patients in Anhui province, China, and that social capital exhibits a stronger association with mental health than physical health. China may not have fully exploited the contribution of social capital, especially social participation, in enhancing QoL. Social capital development policy warrants further consideration.

## Supporting Information

Table S1 **Social capital dimensions and items.**
(DOC)

Table S2 **Mean scores and percentiles for the MOS-HIV questionnaire domains.**
(DOCX)
