# Supplementary material for: Impact of Individual-Level Social Capital on Quality of Life among AIDS Patients in China
Source: PLoS One. 2012 Nov 6;7(11):e48888. doi: 10.1371/journal.pone.0048888 (PMC3490922; doi:10.1371/journal.pone.0048888)
Supplement: Table S1 — Social capital dimensions and items. (DOC) [file pone.0048888.s001.doc]

**TableS1. Social capital dimensions and items.**

| Dimensions | Items |
| --- | --- |
| Social network and ties | 1. How many intimate relatives do you have? |
|  | 2. How many close friends do you have? |
|  | 3. How often do you visit your neighbors? |
|  | 4. How often do you invite your neighbors to your home? |
| Social support | 5. Can you get the care when you feel uncomfortable or are suffering from the disease flare-ups? |
|  | 6. Can you get financial assistance when you experience family life difficulties? |
|  | 7. Do you believe that if you have private problems, you can discuss them with residents in your community? |
|  | 8. Who could you turn to for support when the above situation occurs? |
| Social participation | 9. How many groups or organizations have participated in? |
|  | 10. How many times have you taken part in the activities held by organizations you have joined? |
|  | 11. How many times have you participated in collective community activities? |
| Reciprocity and trust | 12. Do you believe that the majority of residents in your community can be trusted? |
|  | 13. Do you believe that the majority of local hospital and CDC staff can be trusted? |
|  | 14. Do you believe that the majority of residents in your community participate in activities organized by the community for the benefit of only a few residents? |
|  | 15. Would you like to provide support for residents in your community who need help? |
