# Supplementary material for: Impact of Individual-Level Social Capital on Quality of Life among AIDS Patients in China
Source: PLoS One. 2012 Nov 6;7(11):e48888. doi: 10.1371/journal.pone.0048888 (PMC3490922; doi:10.1371/journal.pone.0048888)
Supplement: Table S2 — Mean scores and percentiles for the MOS-HIV questionnaire domains. (DOCX) [file pone.0048888.s002.docx]

**TableS2. Mean scores and percentiles for the MOS-HIV questionnaire domains.**

| Domain | Mean | SD | Percentile | | |
| --- | --- | --- | --- | --- | --- |
|  |  |  | 25th | 50th | 75th |
| Overall Physical Health summary score (PHS) | 50.13 | 9.90 | 44.16 | 53.75 | 57.78 |
| Overall Mental Health summary score (MHS) | 41.64 | 11.68 | 32.43 | 43.00 | 51.38 |
| **Dimension** |  |  |  |  |  |
| General Health Perception(GHP) | 44.17 | 23.02 | 25.00 | 45.00 | 65.00 |
| Pain(P) | 85.55 | 23.28 | 67.50 | 100.0 | 100.00 |
| Physical Function(PF) | 87.49 | 15.79 | 83.33 | 91.67 | 100.00 |
| Role Functioning(RF) | 75.97 | 37.03 | 50.00 | 100.00 | 100.00 |
| Social functioning(SF) | 55.92 | 21.01 | 50.00 | 50.00 | 75.00 |
| Mental Health(MH) | 58.30 | 22.90 | 36.00 | 64.00 | 76.00 |
| Energy(E) | 54.47 | 26.08 | 30.00 | 60.00 | 75.00 |
| Health Distress(HD) | 63.71 | 25.71 | 40.00 | 65.00 | 85.00 |
| Cognitive Functioning(CF) | 77.79 | 22.11 | 60.00 | 85.00 | 100.00 |
| Quality of Life(QoL) | 47.26 | 21.03 | 25.00 | 50.00 | 50.00 |
| Health Transition(HT) | 46.73 | 12.82 | 50.00 | 50.00 | 50.00 |
